# Olive Leaf Polyphenols Attenuate the Clinical Course of Experimental Autoimmune Encephalomyelitis and Provide Neuroprotection by Reducing Oxidative Stress, Regulating Microglia and SIRT1, and Preserving Myelin Integrity

**DOI:** 10.1155/2020/6125638

**Published:** 2020-07-30

**Authors:** Jasminka Giacometti, Tanja Grubić-Kezele

**Affiliations:** ^1^Department of Biotechnology, University of Rijeka, Radmile Matečić 2, 51000 Rijeka, Croatia; ^2^Department of Physiology and Immunology, Faculty of Medicine, University of Rijeka, Braće Branchetta 20, 51000 Rijeka, Croatia; ^3^Clinical Department for Clinical Microbiology, Clinical Hospital Center Rijeka, Krešimirova 42, 51000 Rijeka, Croatia

## Abstract

Numerous evidences suggest that plant polyphenols may have therapeutic benefits in regulating oxidative stress and providing neuroprotection in many neurodegenerative diseases, including multiple sclerosis (MS). However, these mechanisms are not yet completely understood. In this study, we investigated the effect of olive leaf polyphenols on oxidative stress through oxidation marker level and activity (TBARS, SOD, and GPX) and their protein expression (SOD1, SOD2, and GPX1), as well as the protein expression of Sirtuin 1 (SIRT1) and microglia markers (Iba-1, CD206, and iNOS) and myelin integrity (proteolipid protein expression) in the brain of rats with induced experimental autoimmune encephalomyelitis (EAE) and subjected to olive leaf therapy. Experiments were performed in male EAE DA rats, which were randomly divided into 2 main groups: EAE groups treated with the therapy of olive leaf (EAE+TOL) and untreated EAE control groups. The EAE treated groups consumed olive leaf tea instead of drinking water (*ad libitum*) from the beginning to the end of the experiment. In addition, olive leaf extract was injected intraperitoneally (*i.p*.) for the 10 continuous days and started on the 8^th^ day after EAE induction. The clinical course was monitored in both groups until the 30^th^ day after EAE induction. Our results demonstrated that TOL attenuated the clinical course of EAE; reduced the oxidative stress (by decreasing the concentration of MDA); upregulated antioxidant enzymes (SOD1, SOD2, and GPX1), SIRT1 (overall and microglial), and anti-inflammatory M2 microglia; downregulated proinflammatory M1 type; and preserved myelin integrity. These data support the idea that TOL may be an effective therapeutic approach for treating MS and other neurodegenerative diseases.

## 1. Introduction

A broad range of evidence suggests that oxidative stress plays a major role in the pathogenesis of neurodegenerative diseases, including multiple sclerosis (MS) [1, 2]. Reactive oxygen species (ROS), which if produced in excess during inflammation lead to oxidative stress, have been implicated as mediators of demyelination and axonal damage in both MS and its animal models.

One of the most studied cell populations in the central nervous system (CNS) in the context of ROS-mediated tissue damage in MS are microglia cells. An activated microglia produce ROS [3] and NO radicals in MS lesions, which suggests their role in the demyelination and neurodegeneration process of MS [4–8] and accounts for the features of MS pathological findings [9].

Oligodendrocyte progenitor cells (OPCs) are particularly vulnerable to oxidative stress because they have lower levels of antioxidant enzymes [8]. The extent of lipid and DNA oxidation correlates significantly with inflammation and oxidative injury of oligodendrocytes and neurons, which is also associated with active demyelination and axonal and neuronal injury [10], together with upregulated expression of oxidative molecules and antioxidant enzymes in MS lesions [11–13].

However, and this aspect is less well understood, the extracellular and intracellular redox milieu is integral to many processes underlying T cell activation, proliferation, and apoptosis and subsequent neuropathological processes. Besides the promotion of demyelination through oxidative damage [14], release of proinflammatory cytokines (IL-1*β*, IL-6, and TNF-*α*), and increased expression of iNOS and ROS [15], microglia contribute to the repair-permissive environment by providing growth factors, such as IGF-1 and FGF-2 [16], and provide myelin debris clearance for adequate oligodendrocyte differentiation and ongoing myelination/remyelination [17–19].

Furthermore, it seems SIRT1, the nicotinamide adenine dinucleotide- (NAD^+^-) dependent deacetylase highly expressed in both neurons and glial cells in the brain [20–22], is a crucial component of multiple interconnected regulatory networks that modulate dendritic and axonal growth, as well as survival against oxidative stress [23].

Moreover, SIRT1 exerts neuroprotective effects in many models of microglial activation-induced neurodegenerative disease [24, 25].

A decrease in SIRT1 levels and activities is related to inflammation-associated diseases, including various neurodegenerative diseases [26, 27]. Moreover, the reduction of SIRT1 expression could contribute to microglial activation and neuroinflammation [28]. Thus, pharmacological activation or upregulation of SIRT1 may be a promising strategy for the treatment of inflammation-related neurodegenerative diseases. Till now, there are already some phytochemical compounds that have been confirmed to have the ability to increase SIRT1 expression and activity, including resveratrol, quercetin, catechin, and protocatechuic acid [29–33].

Numerous evidences reported that olive leaf phenolics have an antioxidant effect [34], and it seems to have a good potential therapeutic effect on the prevention of neurodegenerative diseases; however, further investigation in humans is needed.

Nevertheless, the effect of olive leaf polyphenols (OLP) on SIRT1 during microglial activation is not completely understood. However, there is little data regarding the effect of OLP in MS or its animal models. Here, we examined the effect of OLP on oxidative stress mediators (SOD and GPX), SIRT1, and microglia and integrity of myelin in experimental autoimmune encephalomyelitis (EAE), an animal model of MS, in an attempt to provide further insights into the neuroprotective potential of olive leaf polyphenols.

## 2. Materials and Methods

### 2.1. Experimental Animals

Experiments were performed on male Dark Agouti (DA) rats, aged 2–3 months. They were housed under standard conditions of light, temperature, and humidity with unlimited access to food and water. Experimental procedures involving animals complied with Croatian laws and rules (NN 135/06; NN 37/13; NN 125/13; NN 055/2013) and with the guidelines set by the European Community Council Directive (86/609/EEC). The experimental protocol was approved by the Ethics Committee of the Department of Biotechnology University of Rijeka (2170-57-005-02-17-1).

#### 2.1.1. EAE Induction and Evaluation

Induction of chronic relapsing- (CR-) EAE was performed in male DA rats by bovine brain white matter homogenate emulsion (BBH) in the complete Freund's adjuvant (CFA) (Sigma, St. Louis, Mo., USA), as previously described [35]. To each animal, 0.1 mL emulsion was injected subcutaneously in each hind footpad. The evaluation of the clinical course was assessed daily using the following criteria: 0: no symptoms; 1: flaccid paralysis of the tail; 2: hind leg paresis; 3: hind leg paralysis with incontinence; and 4: the death of the animal.

#### 2.1.2. Preparation of the Olive Leaf Tea and Olive Leaf Extract (OLE)

Olive leaf extract (OLE) was prepared from samples of olive tree leaves according to the method of Giacometti et al. [36]. The dry residue was weighed and then dissolved in sterile saline and kept at -20°C until its use as therapy together with olive leaf tea (olive leaf therapy; TOL). In addition, the sample of OLE was analyzed using ultrahigh-pressure liquid chromatography with a diode array detector (UHPLC-DAD) in order to determine the concentration of oleuropein and other major phenolics in the extract according to the method of Giacometti et al. [36]. The administrated dose of olive leaf extract (OLE) was 1024 mg/kg, while the concentration of oleuropein was 45.96 mg/kg.

Olive leaf tea was prepared from the dry and ground olive leaf by pouring hot water (at 60-80°C) over plant matter (1.5%, *w*/*v*) and infused for 30 min. After then, the plant matter was removed by filtration. Analysis of major phenolics in the water infusion was performed using the UHPLC-DAD method according to the method of Giacometti et al. [36]. The concentration of oleuropein in tea was 1.5 mg/mL.

### 2.2. Experimental Design

EAE rats were randomly divided into 2 main groups: EAE groups treated with the therapy of olive leaf (EAE+TOL) and untreated EAE control groups. Both main groups are divided into 2 smaller groups sacrificed on different days after EAE induction, i.e., on the 20^th^ day or the 2^nd^ relapse (EAE+TOL 20d and EAE 20d group) and the 30^th^ day or the 2^nd^ remission (EAE+TOL 30d and EAE 30d group). The experimental groups (EAE+TOL 20d and EAE+TOL 30d) were treated with olive leaf tea *ad libitum* from the first day after EAE induction and with OLE injected intraperitoneally (*i.p.*) for 10 continuous days starting from the 8^th^ day after EAE induction (a day before the onset of the first EAE symptoms). This study design was chosen as the design with the highest amelioration of the clinical course symptoms of EAE from the pilot experiments conducted with different olive leaf polyphenol concentrations (see Suppl. Figure 1). Control EAE groups (EAE 20d and EAE 30d) were treated in the same way with physiological saline solution. The fifth group of rats was untreated or did not get EAE induction nor treatment with olive leaf therapy (untreated). EAE rats were sacrificed by exsanguination on the 20^th^ day after induction (*n* = 5) and on the 30^th^ day after induction (*n* = 5). EAE rats treated with OLE were sacrificed on the same days as untreated EAE rats (on the 20^th^ and 30^th^ day after EAE induction, *n* = 5 each). The exsanguination was done in deep anaesthesia (EAE, EAE+TOL, and untreated rats), induced by a combination of ketamine (80 mg/kg) and xylazine (5 mg/kg), given by intraperitoneal (i.p.) injection, according to the guidance of European Community Council Directive (86/609/EEC) and recommendation of the National Centre for the Replacement, Refinement and Reduction of Animals in Research (http://www.nc3rs.org.uk).

### 2.3. Tissue Preparation for Paraffin Slices

The rat brain hemisphere samples were fixed in 4% buffered paraformaldehyde (Sigma-Aldrich, St. Louis, MD, USA) solution during 24 h. Tissue was then embedded in paraffin wax, and sections were cut at 4 *μ*m using the HM 340E microtome (Microtome, Germany). Heat-induced epitope retrieval was done prior to staining procedure by heating tissue slides in boiled citrate buffer pH 6.0 for four times, each 5 min, using a microwave steamer.

### 2.4. Immunohistological and Immunofluorescence Staining

#### 2.4.1. Immunohistochemistry

Immunohistochemical labeling of proteolipid protein (PLP) was performed on paraffin-embedded tissues using DAKO EnVision+ System, Peroxidase (DAB) kit, according to the manufacturer's instructions (DAKO Cytomation, USA), as previously described [36, 37]. Briefly, slices were incubated with peroxidase block to eliminate endogenous peroxidase activity.

After washing, rabbit polyclonal anti-myelin PLP IgG antibodies (Abcam, UK, diluted 1 : 1000 with 1% BSA in PBS) were added to tissue samples and incubated overnight at 4°C in a humid environment, followed by 45 min incubation with peroxidase-labeled polymer conjugated to goat anti-mouse or anti-rabbit immunoglobulins containing carrier protein linked to Fc fragments to prevent nonspecific binding. The immunoreactions' product was visualized by adding substrate chromogen (DAB) solution. Tissues were counterstained with hematoxylin, dehydrated through graded ethanol, and mounted using Entellan (Sigma-Aldrich, Germany). The photomicrographs were taken and examined under an Olympus BX51 light microscope (Olympus, Japan).

#### 2.4.2. Immunofluorescence

Immunofluorescence labeling was also performed on paraffin-embedded tissue sections. Nonspecific binding was blocked by one-hour incubation with 1% BSA in PBS containing 0.001% NaN_3_ at room temperature, as previously described [37]. The following primary antibodies were used: rabbit polyclonal anti-SIRT1 IgG (GeneTex, Alton Pkwy, Irvine, CA, USA, 1 : 200), goat polyclonal anti-Iba-1 IgG (Abcam, Cambridge, UK, 1 : 200), rabbit polyclonal anti-iNOS IgG (Abcam, Cambridge, UK, 1 : 200), and rabbit polyclonal anti-CD206/Mannose receptor IgG (Abcam, Cambridge, UK, 1 : 200). Primary antibodies were diluted in blocking solution and incubated with tissue sections overnight at 4°C in a humid environment. To visualize immunocomplexes, the following secondary antibodies were used: Alexa Fluor donkey anti-rabbit IgG 594 nm (Molecular Probes, Carlsbad, CA, USA, 1 : 500) and Alexa Fluor donkey anti-goat IgG 488 nm (Molecular Probes, Carlsbad, CA, USA, 1 : 300). Secondary antibodies were diluted in blocking solution and incubated with tissue sections in the dark for 1 h at room temperature in a humid environment. Nuclei were visualized by DAPI staining (1 : 1000 in PBS for 5 min; Molecular Probes, Carlsbad, CA, USA). Slides were afterwards washed in PBS and mounted with Mowiol (Sigma-Aldrich, Germany). The photomicrographs were taken under a fluorescent microscope equipped with DP71CCD camera (Olympus, Japan) and Cell F imaging software.

### 2.5. Immunohistochemical Staining Quantification and Cell Counting

#### 2.5.1. Quantification

The immunohistochemical staining quantification of protein expression was performed on 4 *μ*m tissue sections from paraffin-embedded tissues of the brain using Cell F v3.1 software (Olympus Soft Imaging Solutions), as previously described [38]. Captured images were subjected to intensity separation. They were subsequently inverted, resulting in grayscale images with different intensity ranges, depending on the strength of immunohistochemical signals. Regions of interest (ROIs) were arranged to cover the area being analyzed, and mean gray values were measured. ROI surface size was always equal for each analyzed area. Twelve ROIs were analyzed per field (400x) on 3 separate microscopic slides of different tissue samples per animal, obtained from 5 animals/group. The data were expressed as the mean gray value ± SD.

#### 2.5.2. Cell Counting

In the dentate gyrus, subventricular zone, and cortex were estimated the SIRT1^+^, iNOS^+^, and CD206^+^ microglial cells by using antibodies and DAPI staining, respectively. SIRT1^+^ and Iba-1^+^ cells were counted manually in an image surface area of 0.014 mm^2^ and magnification of 1000x. Iba-1^+^ CD206^+^ and Iba-1^+^ iNOS^+^ cells were counted manually in an image surface area of 0.053 mm^2^ and magnification of 400x. Results are expressed as a mean number of cells per mm^2^ [39].

### 2.6. Tissue Preparation and Homogenization for Western Blot and Biochemical Analyses

Brain tissue for protein isolation was obtained from 5 intact rats (untreated), 10 EAE rats (5 EAE 20d and 5 EAE 30d), and 10 EAE rats treated with olive leaf therapy (5 EAE+TOL 20d and 5 EAE+TOL 30d). The rat brain hemisphere samples were rapidly removed and snap-frozen in liquid nitrogen for protein isolation and stored at -80°C. Bead Ruptor 12 homogenizer (Omni International, Kennesaw GA, USA) was used for the preparation of brain tissue homogenates (10% *w*/*v*) from frozen samples. Briefly, one rat cerebral hemisphere was placed into 7 mL tube containing 1.4 mm ceramic beads and suspended with 100 mM Tris-HCl buffer pH 7.6 containing phosphatase and protease inhibitors. The samples were processed at speed 4 for three cycles; each was 15 sec with Dwell of 30 sec. The homogenates were then centrifuged in an Eppendorf 5427R centrifuge (Eppendorf, Hamburg, Germany) for 10 min at 5000 rpm and 4°C. The obtained supernatants were aliquoted and stored at -80°C until analysis. Protein concentrations in supernatants of brain homogenate were determined according to the manufacturer procedure using a BCA protein assay kit (Pierce, Thermo Scientific, Rockford, IL, USA).

### 2.7. Biochemical Analyses

#### 2.7.1. Determination of Malondialdehyde

The lipid peroxidation level (as TBARS) was measured spectrophotometrically by the estimation of malondialdehyde concentration (nmol/mg of proteins) based on the reaction with thiobarbituric acid (TBA) according to the modified method by Ohkawa et al. [40]. Briefly, 100 *μ*L of brain tissue homogenate supernatant was added in the test tube that contained the mixture of 1% *w*/*v* of TBA dissolved in 10% *w*/*v* trichloroacetic acid (TCA) and 2% *w*/*v* butylhydroxytoluene (BHT) dissolved in 10% *w*/*v* TCA. The test tubes were kept for boiling at 90°C for 20 min. After cooling, the tubes were centrifuged at 10000 rpm for 15 min at RT. Separated supernatant was collected and absorbance read at 532 nm using Eppendorf BioSpectrometer® basic (Eppendorf AG, Hamburg, Germany) against reagent blank. All absorbances were read in triplicate. 1,1,3,3-Tetraethoxypropane (TEP) was used as a standard for calibration curve in the range of 0 to 125 *μ*M (*y* = 0.019*x* + 0.0102, *R*^2^ = 0.99996).

#### 2.7.2. Glutathione Peroxidase Activity

Glutathione peroxidase (GPX) activity was determined in the brain homogenate supernatants by commercial Ransel kit (Randox, Crumlin, UK) according to the manufacturer's instructions. GPX activity was calculated as U/mg protein/min.

#### 2.7.3. Superoxide Dismutase Activity

The total superoxide dismutase (SOD) activity was determined in the supernatants of brain homogenates using Ransod kit (Randox, Crumlin, UK) according to the manufacturer's instructions. The percent of SOD inhibition was found between 63.98 and 95.36% (*y* = 43.238*x* + 63.984, *R*^2^ = 0.9929). SOD activity was calculated in terms of U/mg protein/min. GPX and SOD activities were measured at room temperature (RT) using BioSpectrometer fluorescence (Eppendorf, Hamburg, Germany).

### 2.8. Western Blot Analysis

The supernatants were collected and used for the determination of SOD1, SOD2, GPX1, SIRT1, Iba-1, and myelin basic protein (MBP). Briefly, 50 *μ*g protein was subjected to SDS-PAGE and transferred to a PVDF membrane using a semidry protocol after previous protein determination by the BCA method. Electrophoretic separation was performed using precast 4–15% TGX gels in the Mini-PROTEAN Tetra Vertical Electrophoresis Cell (Bio-Rad, Hercules, CA, USA) according to the manufacturer's procedure. The transfer run was at 275 mA for 30 min in an SD10 semidry blotter (Cleaver Scientific Ltd., Rugby, Warwickshire, UK). The membranes were blocked in TBST with a 5% *w*/*v* nonfat dry milk, incubated with primary rabbit monoclonal antibodies SOD1 (Booster Biological Technology, Pleasanton, CA, 1 : 1000), SOD2 (Booster Biological Technology, Pleasanton, CA, 1 : 1000), GPX1 (Booster Biological Technology, Pleasanton, CA, 1 : 1000), SIRT1 (Cell Signaling, Leiden, Netherlands, 1 : 1000), MBP (Booster Biological Technology, Pleasanton, CA, 1 : 1000), and goat polyclonal Iba-1 antibody (Abcam, Cambridge, UK, 1 : 1000) overnight at 4°C without agitation. After that, membranes were washed five times for 10 minutes with TBST (containing 0.1%, *v*/*v* Tween-20) with agitation and incubated for 2 h at room temperature with the appropriate secondary antibody (peroxidase-conjugated goat anti-rabbit IgG, Booster Biological Technology, Pleasanton, CA, USA, 1 : 2000), with agitation. Next, they were washed again with TBST, five times for 10 min at room temperature. Protein loading was controlled using a monoclonal rabbit antibody against *β*-actin (Cell Signaling, Leiden, Netherlands, 1 : 1000). Chemiluminescent substrate for Horseradish Peroxidase- (HRP-) labeled reporter molecules (Roti®-Lumin, Carl Roth GmbH+ Co. KG) was used for protein detection. The light was detected after then using Image Quant LAS 500 chemiluminescence CCD camera (GE Healthcare UK Ltd., Buckinghamshire, HP7 9NA, UK). The bands were examined densitometrically using ImageJ, an image analysis system (National Institutes of Health, Bethesda, USA) which evaluated the relative amount of protein staining and quantified the results in terms of density. The results of treatment with olive leaf EAE+TOL 20d and EAE+TOL 30d were normalized related to the EAE 20d and EAE 30d, respectively.

### 2.9. Statistical Analysis

The data were evaluated with Statistica (data analysis software system), version 13 (TIBCO Software Inc., 2017, Palo Alto, CA, USA). The distribution of data was tested for normality using the Kolmogorov–Smirnov test. Differences between groups were assessed with either one-way analysis of variance (ANOVA) followed by the post hoc Scheffé test or Mann–Whitney *U* test. For evaluation of death frequency between the EAE and EAE+TOL groups, we used Fisher's exact test. Pearson correlation (*r*) was used for determining the association between Iba-1 and SIRT1 cerebral protein expressions within immunofluorescence images.

The data are expressed as mean ± SD and the level of significance is set at *p* < 0.05.

## 3. Results

EAE was induced in genetically susceptible DA rats, which were then treated with olive leaf therapy. The clinical course and the expression profiles of the following proteins (SIRT1, Iba-1, iNOS, CD206, PLP, SOD1, SOD2, and GPX1), biochemical activity (GPX and SOD), and peroxidation level (as TBARS) were examined in the brain, and the data were compared with the findings in untreated EAE rats and untreated control rats.

### 3.1. Olive Leaf Therapy Attenuates the Clinical Course and Reduces Death Frequency during EAE

The EAE control group of rats (EAE 30d; *n* = 16) and EAE rats treated with olive leaf (EAE 30d+TOL; *n* = 16) were monitored daily during the period of 30 days to evaluate the effects of therapy of olive leaf on the clinical course of the disease.

**Figure 1:**
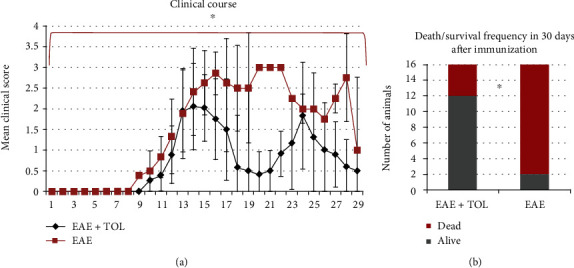
Clinical course and death frequency in the EAE and EAE+TOL groups. (a) The clinical course in the EAE (*n* = 16) and EAE+TOL (*n* = 16) rat groups. Values are presented as mean ± SD (Mann–Whitney *U* test) using EAE scores of each animal for every day. (b) Death/survival frequency during 30 days after EAE induction. Values are presented as a number of animals per group (Fisher's exact test); ^∗^*p* < 0.05.

The intensity of the clinical course of EAE was attenuated in a group of animals treated with olive leaf (EAE+TOL) (Figure 1(a)). The onset of clinical symptoms in the EAE+TOL group was one day after the onset in the EAE group. Furthermore, the clinical scores/symptoms were rising faster in the EAE group during the first five days with a maximum mean score of 3.0 ± 0.0 that reached at the peak of disease. The maximum mean score in the EAE+TOL group that reached at the peak of disease was 2.1 ± 0.8. The first relapse in the EAE group lasted for 3 days with a minimum mean score of 2.5 ± 0.0, unlike the relapse in the EAE+TOL group that lasted for 7 days with a minimum mean score of 0.4 ± 0.5 (Figure 1(a)). During the 30 days after immunization, death occurred in 14 from 16 rats in the EAE group, unlike in the EAE+TOL group, where only 2 from 16 rats died, with a significant difference of *p* = 0.011 (Figure 1(b)).

### 3.2. Biochemical Analysis

**Figure 2:**
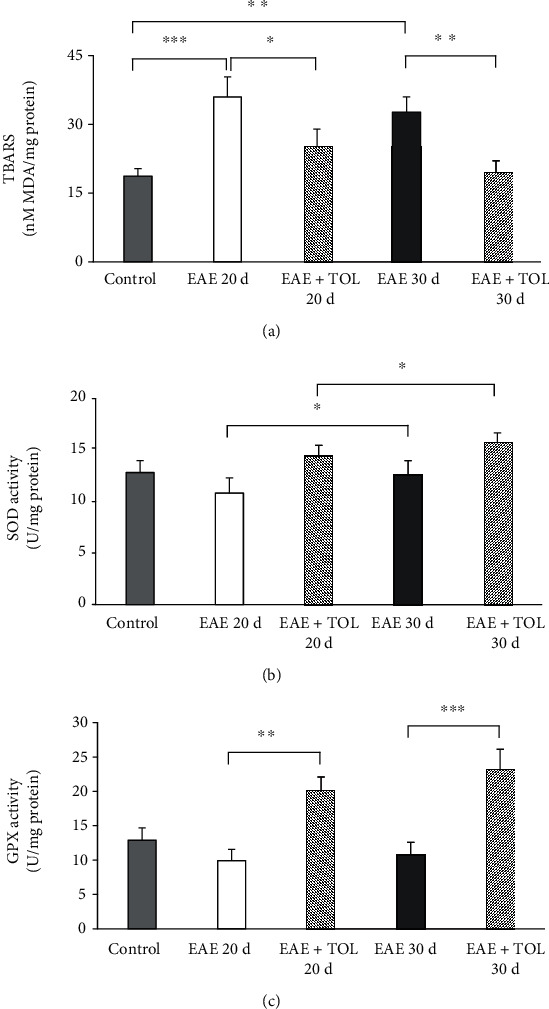
Biochemical assays in the rat brain. (a) The concentration of MDA (nM/mg protein), (b) the activity of SOD (U/mg protein), and (c) the activity of GPX (U/mg protein) in the healthy untreated group (control), in the groups induced EAE after 20 days (EAE 20d) and 30 days (EAE 30d) postimmunization and EAE groups with olive leaf therapy (EAE+TOL 20d and EAE+TOL 30d). For each group, values are presented as the mean ± SD of five rats per group. One-way ANOVA followed by the post hoc Scheffé test was used for the statistical analysis: ∗*p* < 0.05, ∗∗*p* < 0.01, and ∗∗∗*p* < 0.001.

TBARS level and SOD and GPX activity are presented in Figure 2. The level of lipoperoxidation as TBARS is expressed as nM/mg protein of MDA (Figure 2(a)). The level of MDA changed significantly (*p* < 0.001) in all examined groups. TBARS were significantly higher in the EAE groups according to the duration of the illness (*p* < 0.001 for EAE 20d and *p* = 0.001 for EAE 30d). Therapy with olive leaf (TOL) in the EAE groups significantly decreased TBARS level (*p* = 0.009 for EAE+TOL 20d and *p* = 0.001 for EAE+TOL 30d).

Total SOD activity changed related to all examined groups and increased in EAE treated groups (EAE+TOL 20d and EAE+TOL 30d) compared to the untreated control (EAE 20d and EAE 30d), but not significantly (Figure 2(b)). As presented, in the EAE 20d postimmunization group, SOD activity was significantly lower than that in the EAE 30d (*p* = 0.043). The same trend was found in the EAE groups treated with olive leaf polyphenols (*p* = 0.033).

Total GPX activity changed significantly related to all examined groups (*p* < 0.001) (Figure 2(c)). The EAE groups treated with olive leaf polyphenols showed that GPX activity increased significantly in the group EAE+TOL 20d compared to the EAE 20d group (*p* < 0.001) as well as in the EAE+TOL 30d group (*p* < 0.001) compared to the EAE 30d group. These results showed that therapy with OLP affected the increase of endogenous antioxidants in the rat brain during EAE and also reduced lipoperoxidation. Our results showed significant alterations in the antioxidant defenses, especially in the second remission. In addition, the differences were found also in relation to the duration of illness, where the application of OLP can be beneficial to long therapies.

### 3.3. Western Blot Analysis of SOD1, SOD2, GPX1, SIRT1, and Iba-1

**Figure 3:**
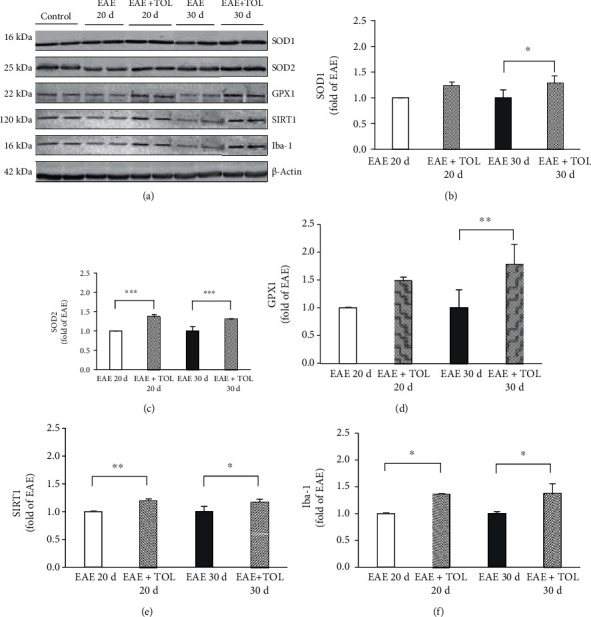
Immunoblot of SOD1, SOD2, GPX1, SIRT1, and Iba-1 in the isolated rat brain proteins. Cell lysate proteins (50 *μ*g) were immunoblotted using *β*-actin as the loading control. (a) Representative western blot images of the target proteins. The expression of (b) SOD1, (c) SOD2, (d) GPX1, (e) SIRT1, and (f) Iba-1 is shown at the normalized expression level of EAE. For each group, values are presented as the mean ± SD of five rats per group. One-way ANOVA followed by the post hoc Scheffé test was used for the statistical analysis: ^∗^*p* < 0.05, ^∗∗^*p* < 0.01, and ^∗∗∗^*p* < 0.001.

Although total SOD activity was lower in the EAE groups and higher in the OLP-treated groups with EAE (Figure 3(b)), we believe that the differences in the protein abundance exist in cytosolic SOD1 (Cu-Zn) and mitochondrial SOD2 (MnSOD) in the rat brain. To determine this alteration, rat brain protein extracts were analyzed with western blot analysis (Figures 3(b) and 3(c)). Therapy with OLP significantly changed cytosolic SOD1 only at the 30^th^ day (*p* = 0.048) and in the mitochondrial SOD2 at both the 20^th^ and 30^th^ days after induced EAE (*p* < 0.001 and *p* < 0.001, respectively). All analyzed groups had the SOD2/SOD1 ratio less than 1.0, after normalizing to the sum of the untreated control group for SOD1 and SOD2, except the EAE+TOL 30d group (the ratio is 1.013). On the other hand, the SOD2/SOD1 ratio was lower in the EAE 20d group (the ratio is 0.814). Thus, we suggested that EAE reduced SOD2 abundance (and activity) in the mitochondria, and OLE therapy enhanced SOD2 in the mitochondria, especially in the remission phase (at 30 days postimmunization). We conclude that EAE progression alters the cytosolic SOD1 and mitochondrial SOD2 protein levels. In addition, OLP therapy effect seems to be compensatory on the mitochondrial SOD2 protein level due to the loss of the specific activity of mitochondrial SOD2 to reduce the deleterious effect of the mitochondrial superoxide.

GPX1 is found in the cytosol and mitochondria to remove a large amount of generated superoxide. We did not find significant changes in the expression of GPX1 in the EAE+TOL 20d group; however, a significant increase of GPX1 expression was found in the EAE+TOL 30d group (*p* = 0.005) (Figure 3(d)).

The western blotting analysis demonstrated that SOD1, SOD2, and GPX1 expressions in OLP-treated EAE rat brain were higher compared to those in EAE animals without the OLP treatment and even higher than those in the untreated control. These data also suggested that the neuroprotective effect of OLP is carried out through its effect on SOD1, SOD2, and GPX1, not only in the acute phase of the disease (20^th^ day) but in the remission period as well (on the 30^th^ day postimmunization). Therefore, the effectiveness of OLP therapy can be proposed, at least in part, to increase levels of these antioxidant enzymes in the brain *in vivo*.

However, the SOD1 and SOD2 protein expression obtained by immunofluorescence revealed a different pattern at the tissue distribution level. Higher SOD1 cell upregulation, including in neurons and microglia, was found in different brain regions (i.e., cortex, hippocampus, and subventricular zone) in rats without the TOL treatment or EAE control rats (see Suppl. Figure 2). Furthermore, the SOD2 expression was upregulated in microglia surrounding lesions in the brain stem from rats without the TOL treatment as well (see Suppl. Figure 3), but not elsewhere.

### 3.4. Olive Leaf Therapy Stimulates Cerebral SIRT1 Expression during EAE

**Figure 4:**
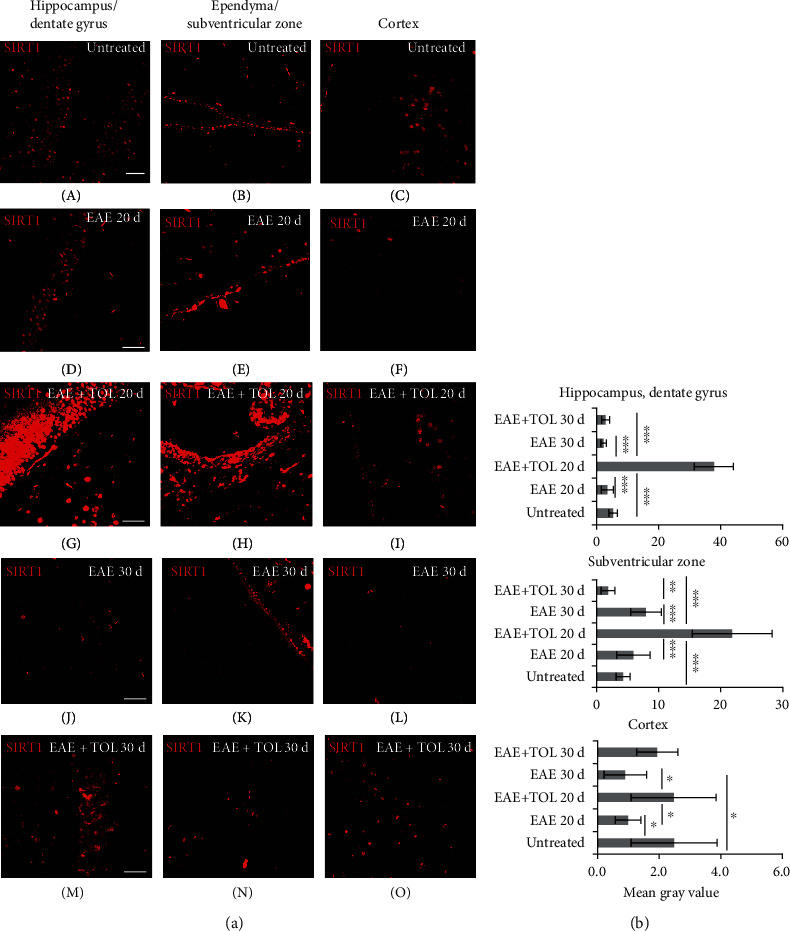
Polyphenols from olive leaf extract induce the upregulation of SIRT1 in different brain regions (hippocampus, ependyma, subventricular zone (SVZ), and cortex). (a) Representative immunofluorescent pictures show staining with anti-SIRT1 antibody in paraffin-embedded sections of the brain tissue obtained from DA rats: (A–C) untreated, (D–F) with induced EAE and the second attack (on the 20^th^ day postinduction), (G–I) with induced EAE and treated with the olive leaf therapy (TOL) till the 20^th^ day postinduction, (J–L) with induced EAE and the second remission (on the 30^th^ day postinduction), and (M–O) with induced EAE and treated with TOL on the 30^th^ day postinduction. (b) SIRT1 immunoreactivity in different brain regions. The immunofluorescent staining quantification was performed using Cell F v3.1 software analysis (12 ROI/4 *μ*m slice × 3 slices/rat × 5 rats/group). Values are expressed as mean gray value ± SD. One-way ANOVA followed by the post hoc Scheffé test: ^∗^*p* < 0.05, ^∗∗^*p* < 0.01, and ^∗∗∗^*p* < 0.001. Scale bars indicate 50 *μ*m.

**Figure 5:**
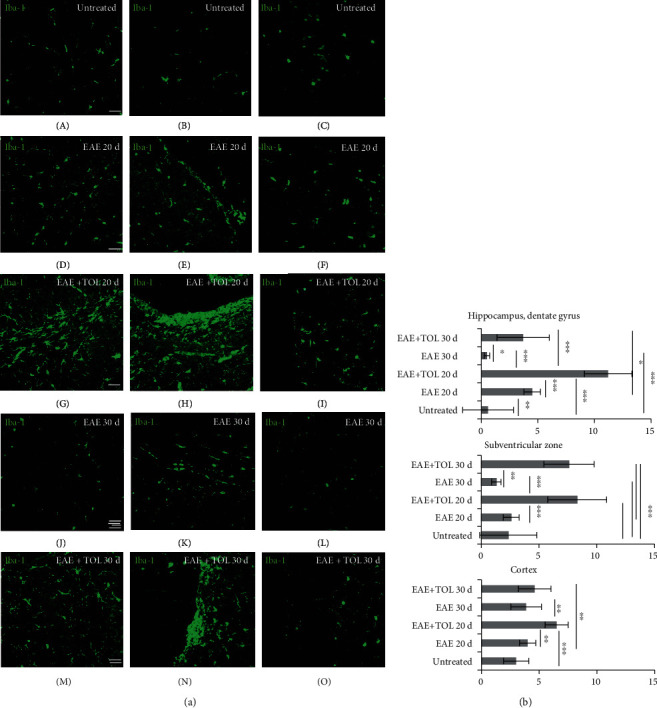
Polyphenols from olive leaf induce the upregulation of Iba-1 in different brain regions (hippocampus, ependyma, subventricular zone (SVZ), and cortex). (a) Representative immunofluorescent pictures show staining with anti-Iba-1 antibody in paraffin-embedded sections of the brain tissue obtained from DA rats: (A–C) untreated, (D–F) with induced EAE and second attack (on the 20^th^ day postinduction), (G–I) with induced EAE and treated with the therapy of olive leaf (TOL) till the 20^th^ day postinduction, (J–L) with induced EAE and the second remission (on the 30^th^ day postinduction), and (M–O) with induced EAE and treated with TOL on the 30^th^ day postinduction. (b) Iba-1 immunoreactivity in different brain regions. The immunofluorescent staining quantification was performed using Cell F v3.1 software analysis (12 ROI/4 *μ*m slice and 3 slices/rat × 5 rats/group). Values are expressed as mean gray value ± SD. One-way ANOVA followed by the post hoc Scheffé test: ^∗^*p* < 0.05, ^∗∗^*p* < 0.01, and ^∗∗∗^*p* < 0.001. Scale bars indicate 50 *μ*m.

Profiling of cerebral SIRT1 protein by immunohistochemistry and western blot showed significant upregulation at the 20^th^ day after EAE induction in rats treated with olive leaf therapy (Figures 3(e) and 4) and greater than that in untreated EAE rats. The data obtained by immunohistochemistry clearly showed that SIRT1 expression was markedly upregulated (Figures 4(a) and 4(b)) in the EAE+TOL 20d group of rats in comparison with findings in the untreated control and untreated EAE group of rats in the following locations: in the hippocampus/dentate gyrus (Figures 4(a) A D, G, J, M and 4(b): *p* < 0.001, *p* < 0.001), in the ependymal, subependymal, and subventricular area (Figures 4(a) B, E, H, K, N and 4(b): *p* < 0.001, *p* < 0.001), and in the cortex (Figures 4(a) C, F, I, L, O and 4(b): *p* = 1.000, *p* = 0.038). The western blot showed, as presented in Figure 5(e), that therapy of olive leaf activated brain SIRT1 in both relapse (20^th^ day) and remission (30^th^ day) phase of the EAE, compared to the same phases in the untreated EAE groups (*p* = 0.009 and *p* = 0.016, respectively). Interestingly, the findings from the relapse phase (20^th^ day) showed higher expression of brain SIRT1 than those from the remission phase (30^th^ day) for normalized values related to untreated control (48% greater for EAE 20d than EAE 30d and 50% greater for EAE+TOL 20d than EAE+TOL 30d) (data not shown). The immunohistochemical findings showed the higher expression level of brain SIRT1 in the relapse phase (20^th^ day) as well than that in the remission phase (30^th^ day), especially in the dentate gyrus (Figures 4(a) M and 4(b); *p* < 0.001) and subventricular zone (Figures 4(a) N and 4(b): *p* < 0.001).

### 3.5. Olive Leaf Therapy Stimulates Cerebral Iba-1 Expression during EAE

To detect the microglia during the olive leaf therapy, a protein marker Iba-1 was used in immunohistochemistry and western blot analyses. The immunohistochemistry showed significant upregulation of Iba-1 on the 20^th^ and 30^th^ days after EAE induction in rats treated with TOL (Figures 5(a) G, H, I, M, N, and O) and greater than that in untreated EAE rats (Figures 5(a) and 5(b)). The data obtained by immunohistochemistry clearly showed that Iba-1 expression was markedly upregulated in the EAE+TOL 20d group of rats in comparison with the findings in the untreated control and untreated EAE group of rats in the following locations: in the hippocampus/dentate gyrus (Figures 5(a) A, D, G, J, and M and 5(b): *p* < 0.001, *p* < 0.001), in the ependymal, subependymal, and subventricular area (Figures 5(a) B, E, H, K, and N and 5(b): *p* < 0.001, *p* < 0.001), and in the cortex (Figures 5(a) C, F, I, L, and O and 5(b): *p* < 0.001, *p* = 0.001). As shown with WB analysis, therapy of olive leaf activated Iba-1 in the EAE 20d (*p* = 0.013) and EAE 30d groups (*p* = 0.010) (*see*Figure 3(f)). Compared to the untreated control, Iba-1 was reduced for 1.8% in the EAE 20d group and 32.3% in the EAE 30d group. In addition, therapy with olive leaf polyphenols enhanced Iba-1 expression for 33% in the EAE+TOL 20d and decreased Iba-1 expression for 6.5% in the group EAE+TOL 30d (data not shown). The immunohistochemical findings showed the higher expression level of Iba-1 marker in the relapse phase (20^th^ day) as well than that in the remission phase (30^th^ day), but only in the dentate gyrus (Figures 5(a) M and 5(b); *p* < 0.001).

#### 3.5.1. Coexpression of Iba-1 and SIRT1 in the Brain

**Figure 6:**
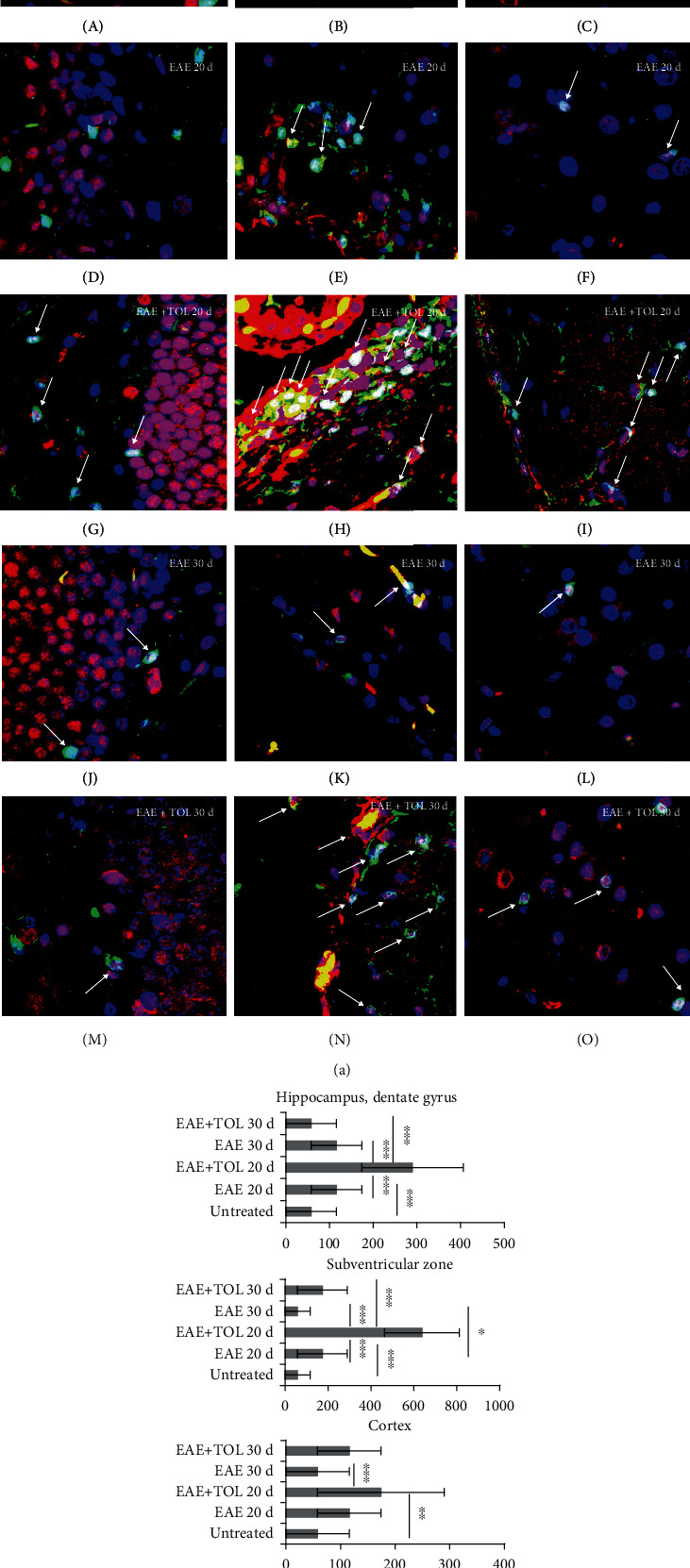
In the cerebral hippocampus, subventricular zone (SVZ), and cortex of rats treated with the therapy of olive leaf (TOL), microglia cells that abundantly express SIRT1 are present. (a) Representative immunofluorescent pictures show the relationship between SIRT1^+^ and Iba-1^+^ microglia cells in DA rats: (A–C) untreated, (D–F) with induced EAE and the second attack (on the 20^th^ day postinduction), (G–I) with induced EAE and treated with TOL till the 20^th^ day postinduction, (J–L) with induced EAE and the second remission (on the 30^th^ day postinduction), and (M–O) with induced EAE and treated with TOL on the 30^th^ day postinduction. (b) A number of Iba-1^+^ SIRT1^+^ cells were manually counted in the area of interest (0.014 mm^2^/4 *μ*m slice × 3 slices/rat × 5 rats/group). Values are expressed as mean gray value ± SD of a number of cells per mm^2^. One-way ANOVA followed by the post hoc Scheffé test: ^∗^*p* < 0.05, ^∗∗^*p* < 0.01, and ^∗∗∗^*p* < 0.001. Scale bars indicate 20 *μ*m.

Immunofluorescent analyses confirmed coexpression of Iba-1 with SIRT1 in the different brain regions (Figure 6), especially in EAE rats treated with the therapy of olive leaf (TOL) (Figures 6(a) G, H, I, N, and O). A number of Iba-1^+^SIRT1^+^ cells per mm^2^ of brain tissue are most abundant in EAE rats treated with TOL till the 20^th^ day after EAE induction (Figure 6(b)), especially in the subventricular zone (Figure 6(a) H, arrows) compared to the untreated EAE 20d (hippocampus: *p* < 0.001; SVZ: *p* < 0.001; and cortex: *p* = 0.081) and control groups of rats (hippocampus: *p* < 0.001; SVZ: *p* < 0.001; and cortex: *p* = 0.039) (Figure 6(b)).

#### 3.5.2. Correlation of Iba-1 and SIRT1 in the Brain

The SIRT1 expression strongly correlated with the Iba-1 expression in the SVZ region (*r* = 0.85, *p* < 0.001), hippocampal region (*r* = 0.85, *p* < 0.001), and cortex region (*r* = 0.61, *p* < 0.001) (Table 1).

### 3.6. Myelin Integrity Is Reduced in EAE Untreated Rats Compared to EAE Rats Treated with Olive Leaf

**Figure 7:**
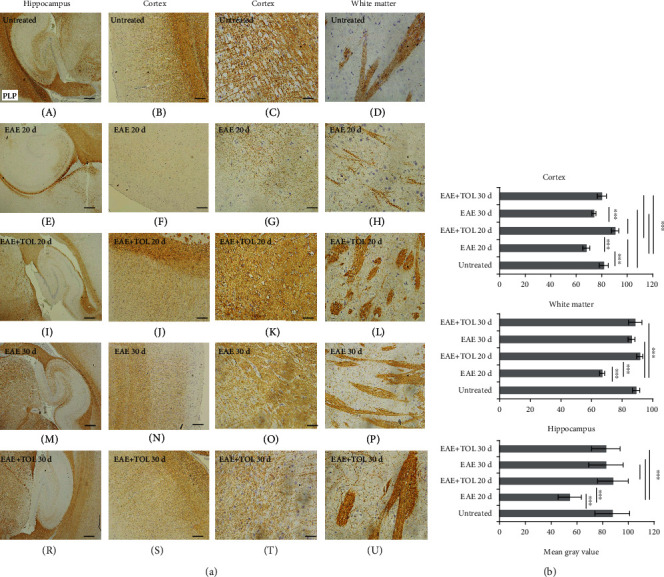
Polyphenols from olive leaf extract induce the upregulation of PLP in different brain regions (hippocampus, cortex, and white matter). (a) Representative immunohistochemical pictures show staining with anti-PLP antibody in paraffin-embedded sections of the brain tissue obtained from DA rats: (A–C) untreated, (D–F) with induced EAE and the second attack (on the 20^th^ day postinduction), (G–I) with induced EAE and treated with polyphenols till the 20^th^ day postinduction, (J–L) with induced EAE and the second remission (on the 30^th^ day postinduction), and (M–O) with induced EAE and treated with polyphenols on the 30^th^ day postinduction. (b) PLP immunoreactivity in different brain regions. The immunohistochemical staining quantification was performed using Cell F v3.1 software analysis (12 ROI/4 *μ*m slice × 3 slices/rat × 5 rats/group) of representative cortex photomicrographs (C, G, K, O, T). Values are expressed as mean gray value ± SE. One-way ANOVA followed by the post hoc Scheffé test: ^∗∗∗^*p* < 0.001. Scale bars in horizontal order indicate 500 *μ*m (for the hippocampus), 200 *μ*m (for the cortex), 50 *μ*m (for the cortex), and 50 *μ*m (for the white matter).

Myelin integrity was assessed by PLP immunostaining which differed significantly between the untreated control group, untreated EAE groups, and EAE groups treated with olive leaf (EAE+TOL) when analyzed (mean gray value ± SD; one-way ANOVA followed by the post hoc Scheffé test PLP; *p* < 0.001). PLP immunostaining was significantly lower in the untreated EAE 20d group vs. the treated EAE+TOL 20d group (*p* < 0.001) and vs. the untreated control group (*p* < 0.001) (Figure 7).

This was accompanied by a corresponding increase in the cellular content of the 18 kDa isoform of MBP at the 30^th^ day compared to the 20^th^ day after inducing EAE (see Suppl. Figure 4B). In addition, MBP expression was significantly greater in rats treated with olive leaf polyphenols at the 20^th^ day of induced EAE. However, we did not find higher MBP after the olive leaf treatment on the 30^th^ day (Suppl. Figure 4C). Since the content of MBP in the brain tissue is a quantitative indicator of the myelin membrane integrity and that myelin also depends on its characteristic lipid contents, therefore, we assume that olive leaf polyphenols attenuate myelin sheath destruction through suppression of the oxidative changes, as well as affect the regulation of lipogenesis. This was supported by the expression of both myelin proteins (PLP and MBP).

### 3.7. Olive Leaf Therapy Influences Microglial M1 to M2 Phenotypic Switch in the Subventricular Zone

**Figure 8:**
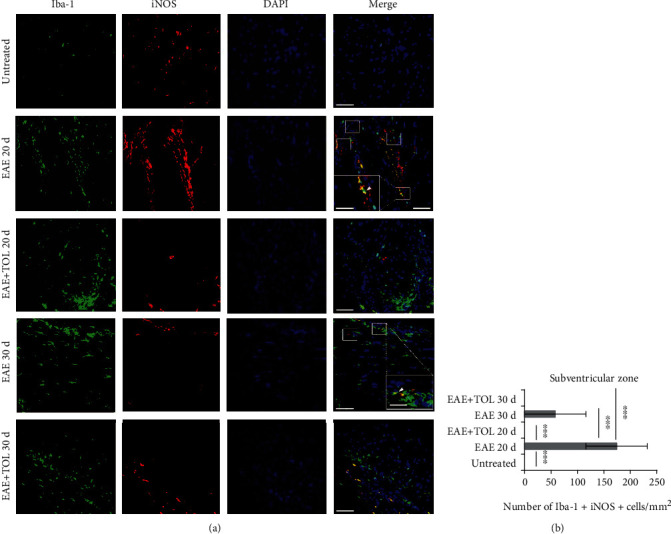
(a) Representative immunofluorescent pictures show the relationship between iNOS^+^ and Iba-1^+^ (M1) microglia cells in the subventricular zone: untreated, with induced EAE and the second attack (on the 20^th^ day postinduction), with induced EAE and treated with TOL till the 20^th^ day postinduction, with induced EAE and the second remission (on the 30^th^ day postinduction), and with induced EAE and treated with TOL on the 30^th^ day postinduction. (b) The number of Iba-1^+^ iNOS^+^ was manually counted in the area of interest (0.053 mm^2^/4 *μ*m slice × 3 slices/rat × 5 rats/group). Values are expressed as mean gray value ± SD of a number of cells per mm^2^. One-way ANOVA followed by the post hoc Scheffé test: ^∗∗∗^*p* < 0.001. Scale bars indicate 50 *μ*m and 20 *μ*m (insets).

**Figure 9:**
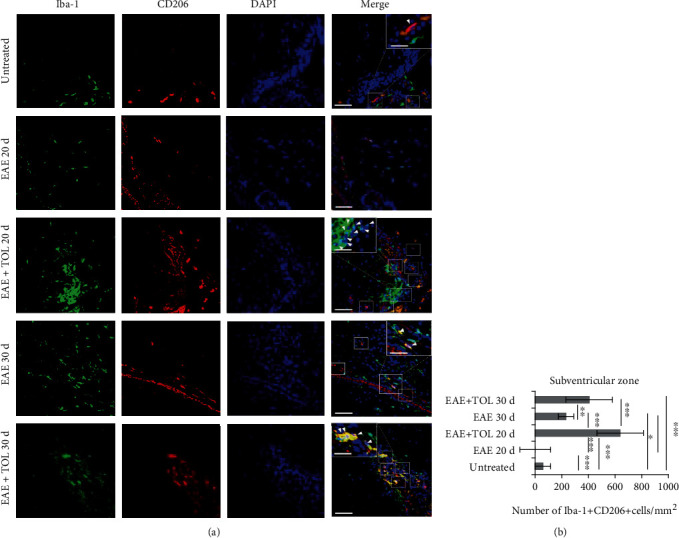
(a) Representative immunofluorescent pictures show the relationship between CD206^+^ and Iba-1^+^ (M2) microglia cells in the subventricular zone: untreated, with induced EAE and the second attack (on the 20^th^ day postinduction), with induced EAE and treated with TOL till the 20^th^ day postinduction, with induced EAE and the second remission (on the 30^th^ day postinduction), and with induced EAE and treated with TOL on the 30^th^ day postinduction. (b) The number of Iba-1^+^ CD206^+^ cells was manually counted in the area of interest (0.053 mm^2^/4 *μ*m slice × 3 slices/rat × 5 rats/group). Values are expressed as mean gray value ± SD of a number of cells per mm^2^. One-way ANOVA followed by the post hoc Scheffé test: ∗*p* < 0.05, ∗∗*p* < 0.01, and ∗∗∗*p* < 0.001. Scale bars indicate 50 *μ*m and 20 *μ*m (insets).

To investigate the effect of olive leaf therapy on microglial M1 and M2 phenotypes, the immunohistochemical staining and number of Iba^+^ CD206^+^ (M2) and Iba^+^ iNOS^+^ (M1) cells in the subventricular zone were determined and compared to the EAE and untreated control groups. TOL significantly decreased the number of microglial M1 cells in the EAE+TOL 20d group compared to both EAE groups (EAE 20d and EAE 30d; *p* < 0.001, *p* < 0.001) (Figures 8(a) and 8(b)). Furthermore, TOL significantly increased the number of microglial M2 cells in the EAE+TOL 20d and EAE+TOL 30d groups compared to both EAE groups (EAE 20d and EAE 30d; *p* < 0.001, *p* = 0.046) and untreated group (*p* < 0.001, *p* < 0.001) (Figures 9(a) and 9(b)). Taken together, these results indicate that olive leaf therapy not only reduces M1 cells but also promotes microglial polarization toward the M2 alternative phenotype.

## 4. Discussion

With this study, we present that therapy with olive leaf polyphenols downregulates the EAE and provides neuroprotection through the attenuation of the clinical course, reduces oxidative stress, regulates microglia and SIRT1, and preserves myelin in the CNS.

Multiple sclerosis (MS) is a chronic inflammatory and neurodegenerative disease of the brain and spinal cord characterized by focal lesions of inflammation, axonal loss, gliosis, and demyelination that affect the white and gray matter [41, 42]. Studies which include EAE, an animal model of MS, have demonstrated that microglia/macrophages actively participate in the pathogenesis of EAE progression [43, 44]. In MS patients, the destruction of myelin in the CNS is associated with activated microglia, which is thought to be involved in the disease pathogenesis [45]. However, other studies indicate that microglia activation counteracts pathological processes by providing neurotrophic and immunosuppressive factors and promoting recovery [46, 47] since the microglia are highly heterogeneous immune cells with a continuous spectrum of activation states [48]. The so-called proinflammatory (M1 phenotype) and the anti-inflammatory (M2 phenotype) microglia are at the opposite ends of this spectrum [49]. The proinflammatory microglia by the activation of T-lymphocytes release proteolytic enzymes, cytokines, oxidative products, and ROS, which affect the development of neurodegeneration in MS. Furthermore, the anti-inflammatory microglia secrete anti-inflammatory cytokines and growth factors that promote oligodendrocyte progenitor proliferation, differentiation, and remyelination and protect neurons from damage [50–53]. Finally, a block in the proinflammatory to anti-inflammatory switch has been hypothesized to contribute to remyelination failure in chronic inactive MS lesions [54]. Thus, our interest is related to the investigations of phytochemicals as possible microglia-targeted therapy for achieving an efficient treatment strategy for MS. As an example for the treatment of relapsing-remitting MS is “Food and Drug Administration-” (FDA-) approved glatiramer acetate which mediates neuroprotective effects by inducing an anti-inflammatory microglial M2 phenotype [55].

However, there are little reports about OLP impact on MS or its animal model EAE. The major bioactive phenolic compound in the olive leaf is oleuropein, while other bioactives are present in the minority [56]. In general, due to the presence of a wide number of bioactive compounds and their synergism, their biological activity is higher than the alone individual.

As described in the study by Miljković et al. [57], the effect of dry olive leaf extract was mediated through the reduction of encephalitogenic cell numbers generated in draining lymph nodes, as well as through inhibition of IFN-g and IL-17 production by the cells infiltrating the CNS.

The main phenolic compound of the OLP extract oleuropein is able to exert, in an indirect way, its antioxidant action by stimulating the expression of intracellular antioxidant enzymes via the activation of nuclear factor erythroid 2-related factor 2 (NrF2) transcription, as well as by increasing the level of nonenzymatic antioxidants [58]. In addition, Park et al. [59] suggested a role for oleuropein in controlling microglial cell activation and as a potential drug candidate for inflammation-mediated neurodegenerative disorders. In our study, we investigated the impact of OLP on microglia with its possible polarization and on the cerebral SIRT1 protein expression. In addition, the impact of OLP on myelin integrity and measure oxidative stress parameters (SOD and GPX activity, lipoperoxidation level as TBARS) was studied as a correlation to antioxidative characteristics of OLP.

Investigating the mechanism of action of natural polyphenols has revealed that they modulate the cell response to oxidative stress via oxidative as well as several other cellular signalling pathways, including regulation of microglia and SIRT1 protein expression [30]. ROS, which if produced in excess lead to oxidative stress, have been implicated as mediators of demyelination and axonal and mitochondria damage as well, in both MS and its animal models [2, 30]. Endogenous antioxidant enzymes can counteract to oxidative stress by conferring protection against oxidative damage. Except for the fact that macrophages and microglial cells exhibit high ROS production in EAE compared to control [60], they produce high levels of superoxide as well in all affected brain areas [61]. Some studies reported that oligodendrocytes are more susceptible to ROS-mediated damage than astrocytes or macrophages [62] due to the high levels of iron found in them, which react with hydrogen peroxide and form the highly toxic peroxynitrite. Next, low levels of glutathione in oligodendrocytes reduce the expression of SOD2 (MnSOD) [63, 64]. Hydrogen peroxide, produced in peroxisomes of oligodendrocytes during the period of active remyelination, affects negatively the long-term repair of myelin and thus causes progression of the disease [65].

Here, we showed that antioxidant enzymes, including SOD1 and SOD2, and GPX1 are markedly downregulated in whole EAE brains compared to normal brain tissue from the control EAE animals. However, by analyzing the tissue distribution of SOD1 and SOD2 proteins, we revealed a different pattern, which is consistent with the study from van Horssen et al. [13, 66]. They reported that antioxidant enzymes, including SOD1 and SOD2, catalase, and heme oxygenase 1, are markedly upregulated in actively demyelinating MS lesions compared to normal-appearing white matter and white matter tissue of the control brains. Hence, they explained that enhanced antioxidant enzymes are due to an adaptive defense mechanism, which reduces ROS-induced cellular damage. Another study reported about impaired antioxidant enzymes in terms of high activity of catalase and decreased activity of MnSOD in peripheral blood mononuclear cells (PBMCs) from patients with relapsing-remitting multiple sclerosis (RRMS) compared to healthy controls [67]. In addition, Stojanović et al. [68] described that in EAE rats, GSH level and SOD activity were decreased in whole encephalitic mass (WEM) and cerebellum homogenates compared to those in untreated control animals.

In our study, the pathological CNS changes in EAE were successfully attenuated by the olive leaf polyphenol application: TBARS were reduced, GPX and SOD activity was enhanced, and in addition, mitochondrial SOD2, cytosolic SOD1, and GPX1 expression levels were increased. Following that, we suggest that the olive leaf polyphenols have the neuroprotective effects and could be successful in the therapy of MS. As reported, SOD2 overexpression, as well as lower immune infiltration, could play a key role in managing underlying pathogenesis of MS and reducing disease severity [69]. Considering that mitochondrial oxidative stress in the CNS affects the modulation of SOD2 function and neurological disorders in neurodegenerative diseases such as stroke, Alzheimer's disease (AD), Parkinson's disease (PD), and age-related loss of cognitive function, we suppose that consumption of olive leaf tea and olive leaf supplements could help in slowing the progression of the disease.

Many studies have already proved the potential ability of natural polyphenols to inhibit and prevent both acute and chronic neurodegenerative diseases, including AD and PD, by decreasing neuronal damage or death [30, 34, 70, 71]. These active compounds are able to modulate cell redox state [72] through the direct action on enzymes, proteins, receptors, and different signalling pathways [73, 74], as well as to interfere with biochemical homeostasis [75, 76]. It has been shown that some of these effects are related to epigenetic modifications of the chromatin [30, 77].

It is described how epigenetic effect of certain cell proteins is due to their ability to deacetylate many transcription factors and thus regulate cell survival, inflammation, and immune function, including neurodegeneration as well [78].

Sirtuin 1 is one such protein, a member of the sirtuin superfamily of histone deacetylases (HDACs), and it is now established that it can directly or indirectly influence the redox property of the cell [23] and reduce oxidative stress through regulation of FOXO3a. The deacetylation of FOXO3a leads to the upregulation of catalase and MnSOD [78]. Sirtuins are NAD^+^-dependent epigenetic and metabolic regulators, which have crucial roles in the physiology of the CNS, immune system, and metabolism. Based on these facts, SIRTs are crucial candidates of therapeutic targets in MS.

Although a large number of studies have focused on sirtuins' functions in health and diseases, the relevance of sirtuins in MS or its animal models is not clear. There are numerous investigations that used disease protective agents and measured SIRT1 level.

Many previous reports of both *in vitro* and *in vivo* studies have proved that natural polyphenols, including those from olive leaf extract, have antioxidant and anti-inflammatory properties *via* modulating important signalling pathways such as NF kappaB, COX-2, and iNOS [29, 79]. Moreover, recently, it was shown that the upregulation of microglial SIRT1, *via* the phenolic compound protocatechuic acid, inhibited the release of inflammatory mediators and ameliorated microglial activation-induced neuron death [29].

The polyphenols are responsible for many health beneficial effects mediated via epigenetic chromatin modifications [78] and can also activate SIRT1 [30]. Therefore, it is possible that the activation of SIRT1 by polyphenols and the resulting inhibitory effects on NF-kappa B and MAPK signalling pathways with a concomitant decrease in release of inflammatory mediators such as TNF-*α* and IL-6 are responsible for the claimed beneficial effect of these compounds including neuroprotection [78–82].

Our results show upregulated SIRT1 in different brain regions of EAE rats treated with olive leaf (Figure 4) (cortex, hippocampus, ependymal, and subventricular zone). However, this upregulation is seen not only in microglia (Figure 6 and Table 1) but also in other cells like neurons, glial, ependymal, and endothelial cells (Figure 4), which is in accordance with other studies investigating SIRT1 expression [83–85]. Still, further investigation on SIRT1 expression in certain cell types in different brain regions is needed for better understanding of its role in proliferation or differentiation during the processes of oligodendrogenesis or neurogenesis.

The data presented in our report are generally in line with the updated knowledge about the antioxidant and anti-inflammatory properties of natural polyphenols, including those from olive leaf [86, 87].

Our hypothesis is based on our findings that SIRT1 is upregulated in microglia and that the most abundant microglia in the brain of EAE rats treated with TOL are anti-inflammatory M2 type or Iba-1^+^ CD206^+^ microglia as seen in Figure 9. These data are shown here only in the subventricular zone as this brain region has gone through the most changes regarding microglial upregulation. In opposite, the subventricular zone had downregulation of proinflammatory M1 type or Iba-1^+^ iNOS^+^ microglia (Figure 8). These data are in high agreement with evidence showing that polyphenols may suppress inflammation mediated by M1 phenotype and influence macrophage metabolism by promoting oxidative pathways and M2 polarization of active macrophages [88].

Microglia may also sense signals from the surrounding environment and have regulatory effects on neurogenesis and oligodendrogenesis in the subventricular zone and the subgranular zone of the hippocampal dentate gyrus [89]. However, which exact signals and cytokines are involved in this process during EAE and during the treatment with OLE should be broadly investigated.

Furthermore, our data showed preserved myelin integrity (Figure 7), which is in correlation with the upregulated SIRT1 and increased superoxide dismutase (SOD) and glutathione peroxidase (GPX) activity found in the brain of EAE rats treated with olive leaf (Figure 2). These findings are supported by already known evidence that SIRT1 antioxidant properties are mediated via modulation of SOD and GPX enzymes [90–93], which prevent the generation of free radicals [94] that cause demyelination during EAE [2].

Clinical signs were coincident with the reduction of MBP in the cortex, while in rats receiving OLP the onset of the disease was delayed and clinical signs were reduced. This amelioration of clinical signs was accompanied by sustained levels of MBP. Busto et al. [95] reported that ellagic acid protects from myelin-associated sphingolipid loss in the cortex in EAE, suggesting a neuroprotective effect. Our results showed that OLP significantly increased 18 kDa MBP at the 20^th^ day, while decreased at the 30^th^ day (Supplementary Figure 4C), but not significantly. We assume that therapy with OLP should be constant throughout the course of the disease to reduce MBP loss. The animals did not receive therapy from the 20^th^ to the 30^th^ day, which reflected on the level of MBP.

Finally, our results demonstrate that TOL attenuates the clinical course of EAE, and the possible mechanisms include reduction of oxidative stress, upregulation of SIRT1 in the brain tissue including microglial cells, upregulation of the anti-inflammatory M2 type of microglia, and preservation of the myelin integrity. These data support the idea that OLE may be an effective therapeutic approach for treating MS and other neurodegenerative diseases as well.

## Figures and Tables

**Table 1:** Pearson correlations (*r*) between Iba-1 and SIRT1 cerebral expressions.

Variable	SIRT1 (SVZ)	SIRT1 (hippocampus)	SIRT1 (cortex)
Iba-1 (SVZ)	0.85∗	/	/
Iba-1 (hippocampus)	/	0.85∗	/
Iba-1 (cortex)	/	/	0.61∗

∗Denotes statistical significance at *p* < 0.05. Iba-1 and SIRT expressions were correlated using the average gray intensity level of immunofluorescent staining.

## Data Availability

The data used to support the findings of this study are available from the corresponding author upon request.

## Supplementary Materials

Supplementary Materials Supplementary Figure S1: pilot experiments for the best study design. Different concentrations or different kinds of administration, i.e., (1) EAE+TEA: only the olive leaf tea ad libitum (oleuropein 1.5 mg/mL) (light blue line); (2) EAE+EXTRACT×3+TEA: olive leaf extract i.p. (45.96 mg/kg 3 times every second day) together with olive leaf tea ad libitum (yellow line); (3) EAE+EXTRACT×10: olive leaf extract i.p. (45.96 mg/kg/day for 10 days) alone (gray line); and (4) EAE+EXTRACT×10+TEA: olive leaf extract i.p. (45.96 mg/kg/day for 10 days) together with olive leaf tea ad libitum (oleuropein 1.5 mg/mL) (orange line). Dark blue line represents EAE without any treatment. The best results or the highest amelioration of clinical symptoms we get with the fourth option or 45.96 mg/kg/day for 10 days i.p. together with olive leaf tea ad libitum and we decided to use it in further investigation (orange line in the figure). The values are presented as mean of EAE scores of each animal for every day. Supplementary Figure S2: in the cerebral hippocampus, subventricular zone (SVZ), and cortex of rats treated with polyphenols from olive leaf extract, microglia cells that express less SOD1 are present. Representative immunofluorescent pictures show relationship between SOD1^+^ cells and Iba-1^+^ microglia cells in DA rats (arrows point the SOD1^+^Iba-1^+^ cells): (a–c) untreated, (d–f) with induced EAE and second attack on the 20th day postinduction, (g–i) with induced EAE and treated with polyphenols till the 20th day postinduction, (j–l) with induced EAE on the 30th day postinduction, and (m–o) with induced EAE and treated with polyphenols on the 30th day postinduction. Scale bars indicate 20 *μ*m. Supplementary Figure S3: representative immunofluorescent pictures show the relationship between SOD2 and Iba-1^+^ microglia cells in the inflamed area of the brain stem: untreated, with induced EAE and the second attack (on the 20th day postinduction), with induced EAE and treated with TOL till the 20th day postinduction, with induced EAE and the second remission (on the 30th day postinduction), and with induced EAE and treated with TOL on the 30th day postinduction. Scale bars indicate 50 *μ*m. Supplementary Figure S4: immunoblot of myelin basic protein (MBP) in the isolated rat brain proteins. Cell lysate proteins (50 *μ*g) were immunoblotted using *β*-actin as the loading control. (A) Representative western blot image of the target protein. (B) The expression of mean MBP is shown at the normalized expression level. (C) The expression of MBP is shown at the normalized expression level of the EAE. For each group, values are presented as the mean ± SD of five rats per group. One-way ANOVA followed by the post hoc Scheffé test was used for the statistical analysis: ∗∗*p* < 0.01 and ∗∗∗*p* < 0.001.
