# Supplementary figures and images for: Olive Leaf Polyphenols Attenuate the Clinical Course of Experimental Autoimmune Encephalomyelitis and Provide Neuroprotection by Reducing Oxidative Stress, Regulating Microglia and SIRT1, and Preserving Myelin Integrity
Source: Oxid Med Cell Longev. 2020 Jul 30;2020:6125638. doi: 10.1155/2020/6125638 (PMC7415106; doi:10.1155/2020/6125638)

Figure s1

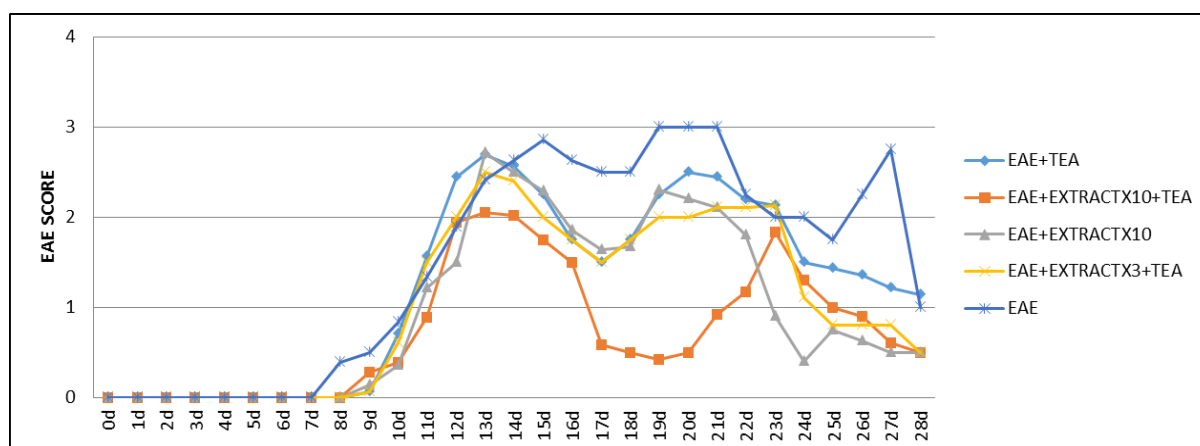

Figure s2

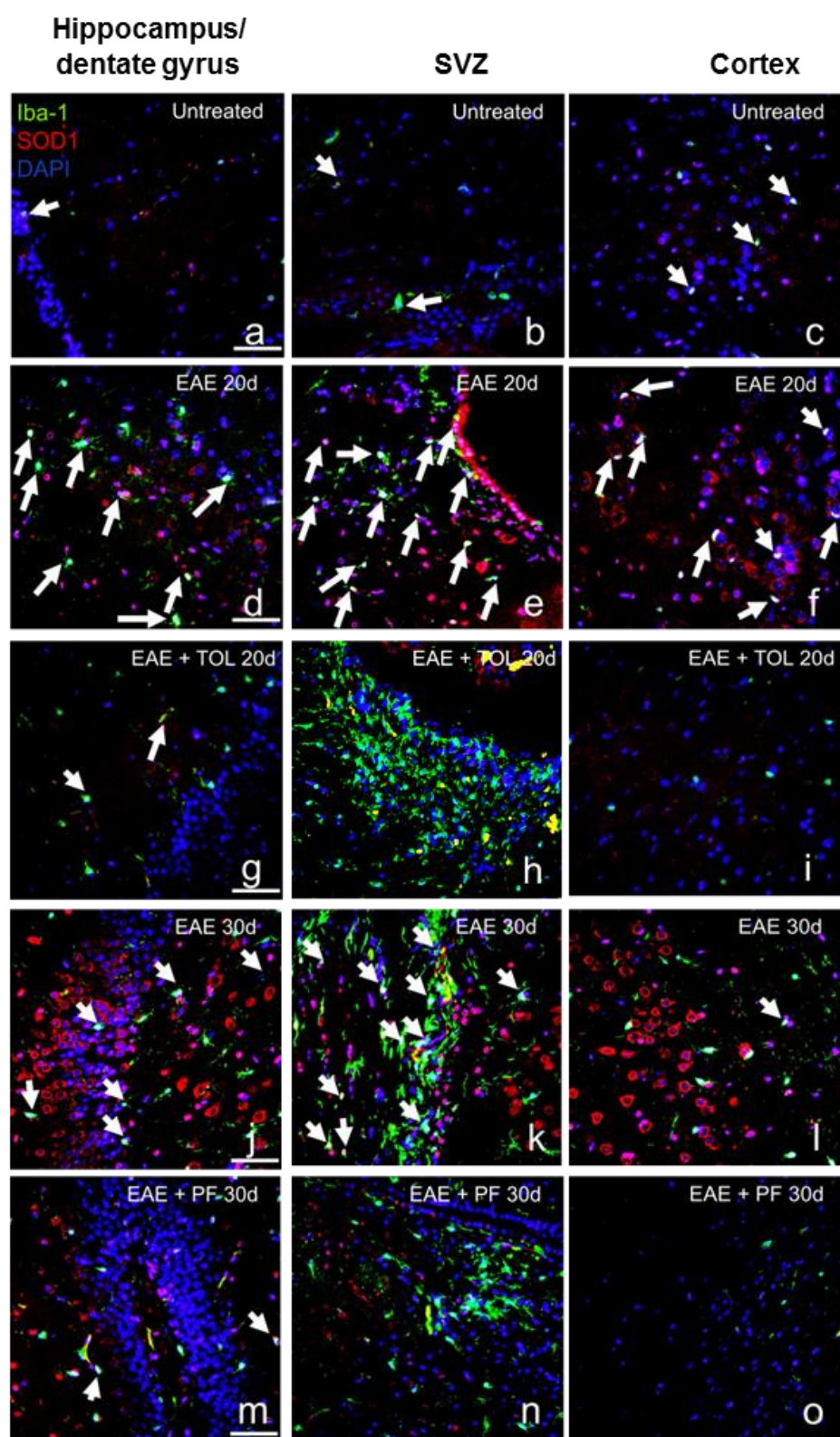

Figure s3

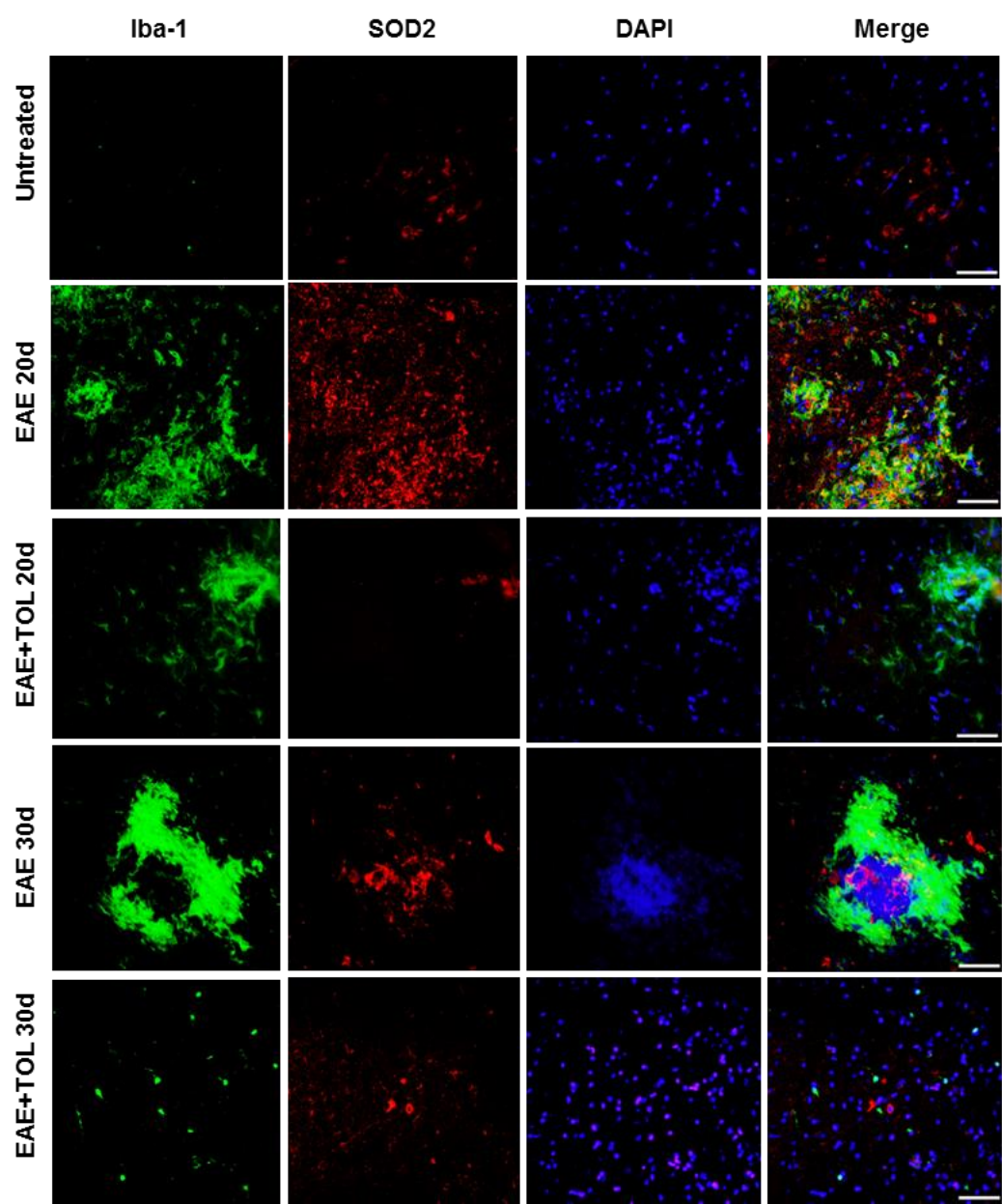

Figure s4

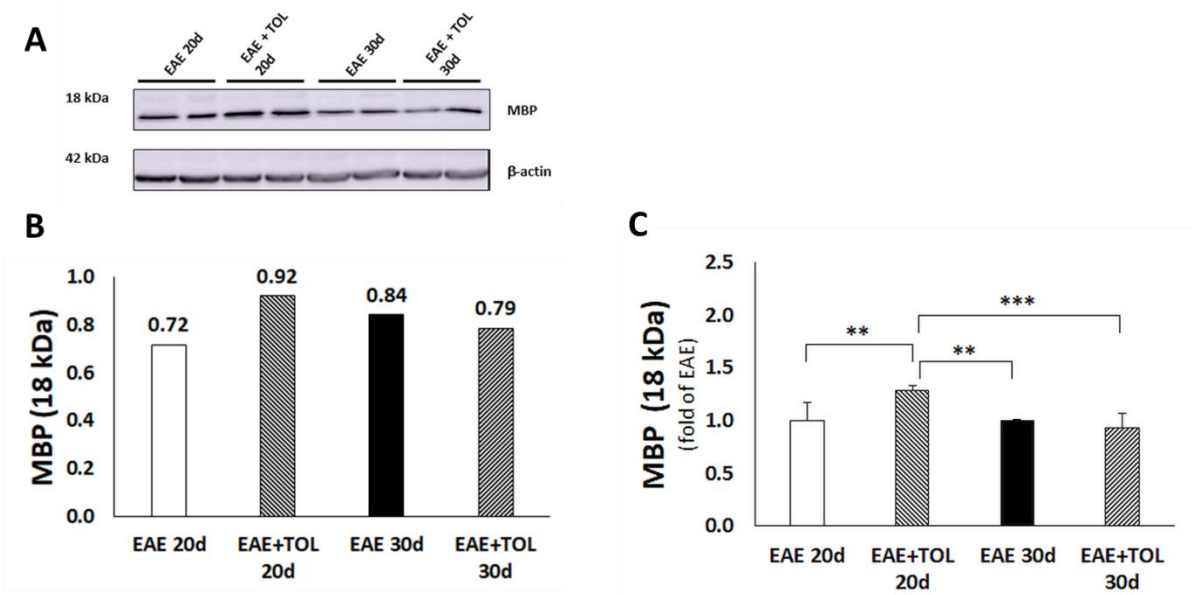

Supplement: Supplementary Materials — Supplementary Figure S1: pilot experiments for the best study design. Different concentrations or different kinds of administration, i.e., (1) EAE+TEA: only the olive leaf tea ad libitum (oleuropein 1.5 mg/mL) (light blue line); (2) EAE+EXTRACT×3+TEA: olive leaf extract i.p. (45.96 mg/kg 3 times every second day) together with olive leaf tea ad libitum (yellow line); (3) EAE+EXTRACT×10: olive leaf extract i.p. (45.96 mg/kg/day for 10 days) alone (gray line); and (4) EAE+EXTRACT×10+TEA: olive leaf extract i.p. (45.96 mg/kg/day for 10 days) together with olive leaf tea ad libitum (oleuropein 1.5 mg/mL) (orange line). Dark blue line represents EAE without any treatment. The best results or the highest amelioration of clinical symptoms we get with the fourth option or 45.96 mg/kg/day for 10 days i.p. together with olive leaf tea ad libitum and we decided to use it in further investigation (orange line in the figure). The values are presented as mean of EAE scores of each animal for every day. Supplementary Figure S2: in the cerebral hippocampus, subventricular zone (SVZ), and cortex of rats treated with polyphenols from olive leaf extract, microglia cells that express less SOD1 are present. Representative immunofluorescent pictures show relationship between SOD1+ cells and Iba-1+ microglia cells in DA rats (arrows point the SOD1+Iba-1+ cells): (a–c) untreated, (d–f) with induced EAE and second attack on the 20th day postinduction, (g–i) with induced EAE and treated with polyphenols till the 20th day postinduction, (j–l) with induced EAE on the 30th day postinduction, and (m–o) with induced EAE and treated with polyphenols on the 30th day postinduction. Scale bars indicate 20 μm. Supplementary Figure S3: representative immunofluorescent pictures show the relationship between SOD2 and Iba-1+ microglia cells in the inflamed area of the brain stem: untreated, with induced EAE and the second attack (on the 20th day postinduction), with induced EAE and treated with TOL till t [file 6125638.f1.pdf]
